# Hybrid Fiber Influence on the Crack Permeability of Cracked Concrete Exposed to Freeze–Thaw Cycles

**DOI:** 10.3390/ma17081819

**Published:** 2024-04-16

**Authors:** Wei Zeng, Weiqi Wang, Qiannan Wang, Mengya Li, Lining Zhang, Yunyun Tong

**Affiliations:** 1 School of Civil Engineering and Architecture, Zhejiang University of Science and Technology, Hangzhou 310023, China; wei-zeng@zust.edu.cn (W.Z.); 121032@zust.edu.cn (W.W.); wangqiannan@zust.edu.cn (Q.W.); 2 Zhejiang International Science and Technology Cooperation Base for Waste Resource Recycling and Low-Carbon Building Materials Technology, Zhejiang University of Science and Technology, Hangzhou 310023, China; 3 Laboratory of Mechanics and Materials of Civil Engineering (L2MGC), EA 4114, CY Cergy Paris Université, Cergy, 95000 Paris, France; mengya.li1@cyu.fr; 4 Department of Mechanical and Mechatronics Engineering, The University of Auckland, Auckland 1010, New Zealand; lzha977@aucklanduni.ac.nz

**Keywords:** crack permeability, hybrid fiber, crack surface, positive synergistic effect, splitting tensile load

## Abstract

This paper describes hybrid fiber’s influence on the crack permeability of cracked concrete exposed to freeze–thaw cycles. A permeability setup and a laser-scanning setup have been designed to measure the crack permeability and the fractured surface roughness of cracked hybrid fiber-reinforced concrete, containing polypropylene fiber and steel fiber, under a splitting tensile load. The results show that, when the effective crack width of the specimens is less than 25 μm, the rough crack surface significantly reduces the concrete’s crack permeability. As the crack width increases, the effect of the concrete crack surface on crack permeability gradually decreases, and the crack permeability of the concrete is closer to the Poiseuille flow model. The permeability parameter *α* derived from the Poiseuille flow model is effective for assessing the crack permeability of concrete. Compared to the modified factor ξ of crack permeability, the permeability parameter α can effectively evaluate and quantify the development trend of crack permeability within a certain range of crack widths. The permeability parameter α of SF20PP2.3, subjected to the same freeze–thaw cycles, decreases by 16.3–94.8% compared to PP4.6 and SF40, and SF20PP2.3 demonstrates a positive synergistic effect on the crack impermeability of cracked concrete. The crack impermeability of SF40PP2.3, subjected to the same freeze–thaw cycles, lies between that of PP6.9 and SF60. The roughness of crack surface (X) and the crack permeability (Y) are highly correlated and follow an exponential curve (Y = 1.0415 × 10^7^·e^−6.025·X^) in concrete. This demonstrates that hybrid fibers enhance crack impermeability by increasing the crack surface roughness. Furthermore, the combination of polypropylene fiber and steel fiber effectively promotes the formation of micro-cracks and facilitates the propagation of multiple cracks in the concrete matrix. This combination increases the head loss of water flow through the concrete and decreases the crack permeability.

## 1. Introduction

In cold regions, concrete structures are usually exposed to freeze–thaw cycles in the service stage. After freeze–thaw cycles, concrete suffers from freeze–thaw damage and its internal micro-cracks increase significantly [1,2]. This increases the permeability of concrete materials. External harmful substances, such as chloride ion and sulfate ion, can easily enter the interior of the concrete structure through micro-cracks caused by freeze–thaw cycles, aggravating the corrosion of the concrete structure and reducing its load-bearing capacity and service life. Generally, the application of macro fibers in civil engineering can effectively reduce internal defects and decrease the concrete damage under harsh environmental conditions such as freeze–thaw cycles [3,4]. The durability of concrete is directly related to its permeability, and concrete structures in the service stage often work with cracks [5,6,7], and the appearance of cracks significantly increases the permeability of concrete [8]. The macro fibers can effectively limit the formation and development of macro-cracks in concrete structures [9,10,11] and decrease the permeability of cracked concrete.

Because the effects of different types of macro fibers on concrete properties are significantly different, the effect of the hybrid application of multiple fibers on concrete has been explored to overcome the limitations of single-fiber reinforced concrete. Soner et al. [12] found that using macro-PP fibers in a hybrid form with micro-PP fibers effectively reduced abrasion loss and improved mechanical capacities more than using single macro-PP fibers. Wang et al. [9] found that fibers with a high elastic modulus can increase the initial cracking stress and ultimate strength, while fibers with a low elastic modulus can effectively increase the toughness and strain capacity of the post-peak behavior of cracked concrete. Athanasia et al. [13] found that hybrid cement–mortar composites, strengthened with high-elastic-modulus carbon fibers and low-elastic-modulus polypropylene fibers, show improved mechanical and electromechanical responses. Therefore, hybrid fiber-reinforced concrete (HFRC) with a different elastic modulus and fibers can effectively show a better performance than concrete reinforced with a single type of fiber. Banthia et al. [14] and Caggiano et al. [15] found that the combination of polypropylene fiber and steel fiber increases the bending–hardening performance of concrete and shows a positive synergistic effect. Afroughsabet et al. [16] found that the combination of polypropylene fiber and steel fiber with a certain ratio can significantly improve the mechanical properties of concrete. Ding et al. [17] found that a hybrid fiber of polypropylene fiber and steel fiber improves the flexural toughness of concrete more than steel fiber or polypropylene fiber alone, with the same volume content. Furthermore, Rashid et al. [18] found that a hybrid fiber of polypropylene fiber and steel fiber enhances the durability performance of concrete structures. It is worth noting that hybrid fibers have a significant effect on concrete properties (including mechanical properties, electromechanical properties and durability properties). In cold regions, the effect of freeze–thaw cycles plays a key role in exacerbating the durability deterioration of cracked concrete structures [19]. So, it is necessary to explore the hybrid fiber (steel fiber and polypropylene fiber) influence on the crack permeability of cracked concrete exposed to freeze–thaw cycles. However, this research work is still very limited.

When the seepage medium and seepage pressure are constant, the crack morphology of concrete significantly impacts the crack permeability of concrete [3]. Previous studies [20,21,22] have shown that the morphology of concrete cracks can often be represented by two-dimensional parameters, such as the tortuosity of cracks on the surface of concrete structures. However, since a concrete crack has three-dimensional characteristics, the two-dimensional parameters do not accurately represent the morphology of concrete cracks. Therefore, this study employs a self-developed 3D laser scanner to scan and construct the crack surface. Based on the laser sensors in the scanner, the coordinates of each point on the concrete crack surface can be effectively measured and the crack surface morphology can be reconstructed, thus quantifying the crack surface roughness. Based on the data of crack surface morphology, a three-dimensional parameter, such as a crack surface roughness number (*R*_n_) [23,24,25], can be calculated, and the hybrid fiber’s influence on the morphology of cracks in cracked concrete can be analyzed.

In engineering practice, the majority of concrete structures serve under loading. The crack morphology under loading significantly differs from that of cracks after unloading. Considering the loading condition, the study investigates the crack permeability of concrete under loads in real time. As the cracks continue to expand, the crack permeability of the concrete increases. Previous studies [26,27,28,29] typically calculated the crack permeability of concrete by quantifying the mass of water flowing out from the water pipes of the permeability setup. However, since the permeability setup includes the specimens, vessels, and water pipes, the process of water flowing out from the water pipes of the permeability setup introduces a delay in the results, or water remaining in the permeability setup also lead to errors in measuring the permeability of cracked concrete. Therefore, this study designs a permeability setup, which makes water flow through the concrete by reducing the air pressure at the downstream of the outflow water from the permeability setup. At the same time, the water mass passing through the concrete is measured by an electronic scale monitoring the water container at the upstream of the permeability setup. The water quantity passing through the concrete is used to calculate the permeability of the concrete. This method reduces the error in measuring the crack permeability and enhances the precision of the results.

Hybrid fibers have been widely used in civil engineering. In order to estimate the effect of the combination of polypropylene fiber and steel fiber on the crack impermeability of concrete structures in engineering applications, a series of tests were carried out: (a) a rapid freeze–thaw test was carried out to introduce freeze–thaw damage to the concrete, (b) the relationships between the crack opening displacement (*D*_COD_) and the effective crack area (*A^eff^*) of the fiber-reinforced concrete (FRC) specimens were estimated, (c) a permeability test under a splitting tensile load was conducted to evaluate the permeability of the concrete and (d) a morphological analysis of the concrete crack surface was conducted, using the developed 3D laser-scanning setup. Furthermore, analyses of the relationship between the crack surface roughness and the permeability parameter *α*, as well as the positive synergistic effect of hybrid fiber on crack impermeability, were conducted. Based on the relationship between the crack surface roughness and the permeability parameter *α* obtained in this study, the permeability of the cracked concrete can be quickly evaluated by the crack surface roughness in real engineering applications. In addition, the permeability–deformation relationship of cracked concrete in this study can be combined with the load–deformation relationship and finite-element analysis of cracked concrete in the service stage. This can effectively estimate the permeability of fiber-reinforced concrete structures under loading and provide a design basis for enhancing the permeability of hybrid fiber (steel fiber and polypropylene fiber)-reinforced concrete under freeze–thaw conditions for engineering applications.

## 2. Experiment

### 2.1. Materials

**Figure 1:**
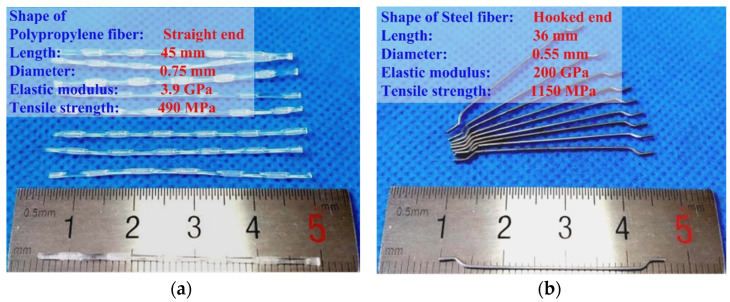
Geometries and performance parameters of macro fibers: (**a**) polypropylene fiber; (**b**) steel fiber.

The mix design of the concrete was as follows: cement (P·O) 390 kg/m^3^, coarse aggregate (5–10 mm) 848 kg/m^3^, fine aggregate (0–5 mm) 822 kg/m^3^, fly ash 155 kg/m^3^, water 272.5 kg/m^3^ and superplasticizer 4.0 kg/m^3^. The macro fibers used include polypropylene fiber and steel fiber. The geometries and performance parameters of the macro fibers are shown in Figure 1. The hybrid fiber consists of polypropylene fiber and steel fiber, and the fiber volume contents of the specimens were 0.50 vol.% and 0.75 vol.%, respectively. The polypropylene fiber-reinforced concrete (PFRC) and steel fiber-reinforced concrete (SFRC), with the same fiber volume content, and normal concrete (NC) were the reference specimens. The specimens were named based on their macro fiber content, as shown in Table 1.

### 2.2. Specimens

**Figure 2:**
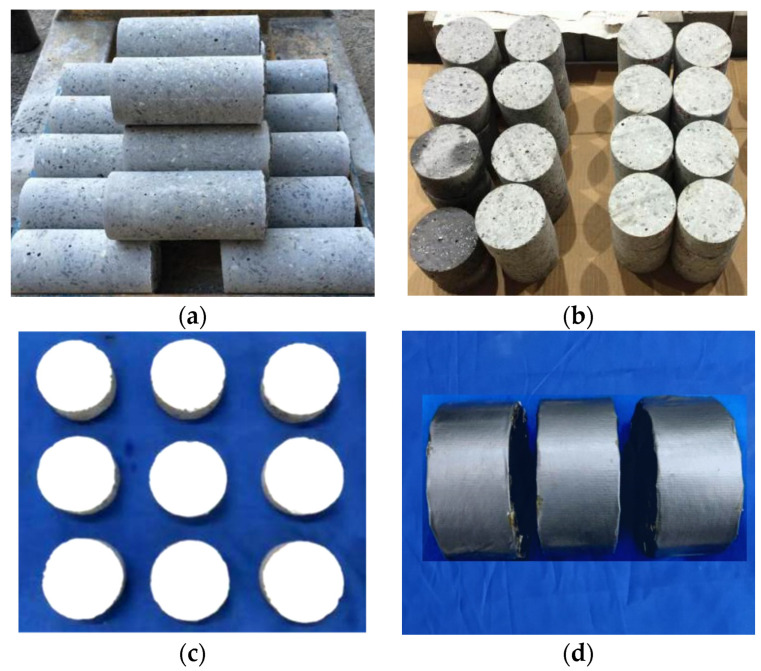
Preparation of cylinder specimen: (**a**) cylindrical specimen (size: diameter = 100 mm, height = 205 mm); (**b**) specimen (size: diameter = 100 mm, height = 50 mm); (**c**) specimen with white color; (**d**) specimen with waterproof tape.

The fresh concrete was poured into steel molds of 400 mm × 400 mm × 205 mm, and it was vibrated for 30 s to remove air bubbles on the vibrating table. A plastic wrap was used to cover the surface to prevent cracking. After demolding, the specimens were cured for 28 days in a standard room at 20 °C and 95% humidity. After curing, a core drill was used to extract cylindrical specimens (size: diameter = 100 mm, height = 205 mm), as shown in Figure 2a. Subsequently, the cylindrical specimens were cut into specimens (size: diameter = 100 mm, height = 50 mm), and the specimens were polished to ensure that their cut surfaces were parallel, as shown in Figure 2b.

A freeze–thaw test was conducted on the specimens, to induce freeze–thaw damage according to ASTM C666 [30]. The target freeze–thaw cycles were 50, 100 and 150, respectively. Twelve specimens were prepared for each specimen. Before the splitting tensile test, the two surfaces of the six specimens were painted white to record the crack patterns on the specimen surfaces, as shown in Figure 2c. Before the permeability tests, the other six specimens were wrapped on the sides with waterproof tape. This prevented water leakage from the sides of the specimens, as shown in Figure 2d.

### 2.3. Acquisition of Crack Data of Concrete Surface under Splitting Tensile Load

**Figure 3:**
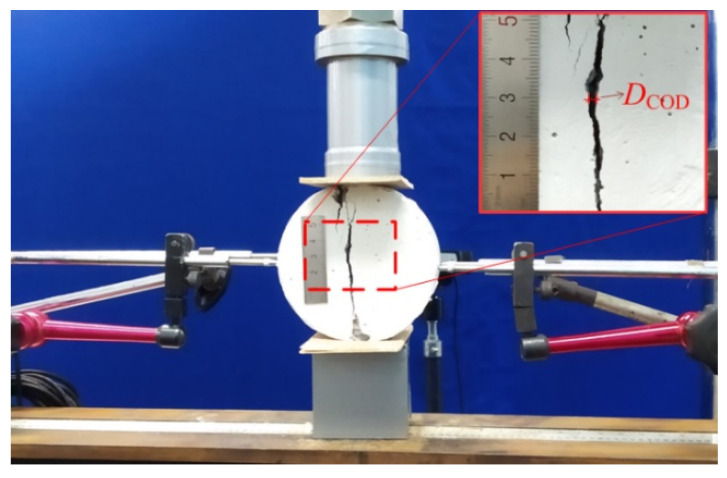
Setup for splitting tensile test.

Splitting tensile tests were conducted on the specimens, following ASTM C496 [31] and GB/T 50081-2002 [32]. An MTS 250 kN hydraulic servo machine (Manufacturer: MTS Systems Corporation (China), Shenzhen, China) with a closed-loop control was used, under a deformation rate of 0.012 mm/min. Two cameras recorded crack patterns in the specimens under loading, as shown in Figure 3.

The surface crack patterns of the concrete specimens were digitized with image processing software, as shown in Table 2. The grayscale tool of the Image Pro Plus software (Image-Pro Plus Version 6.0) initially identified the crack areas and removed non-crack areas. Subsequently, the crack dimensions and areas were calculated with features of the Image Pro Plus software. From Table 2, when the crack opening displacement (*D*_COD_) was small, multiple discontinuous cracks formed on the concrete surface and the separation of the crack surfaces was incomplete, which was caused by the degree of heterogeneity of the concrete [33]. With the increasing of the *D*_COD_, a continuous main crack gradually formed on the concrete surface.

Wang et al. [34] found that concrete was a multiphase heterogeneous material, and cracks typically first appear on one side of the concrete specimen and then extend to the other side. In order to assess the width of crack propagation, the study employed 4 linear variable differential transformers (LVDTs) (Manufacturer: Shenzhen Milont Technology Co., Ltd., Shenzhen, China) arranged on both sides of the specimen along its central axis. The displacement values *d*_1_, *d*_2_, *d*_3_, and *d*_4_ were measured using the four LVDTs, respectively. The radial deformation (diameter variation, *D*_V_) of the specimens under a splitting tensile loading was calculated by Equation (1).

(1) $$D_{V}=\frac{d_{1}+d_{2}+d_{3}+d_{4}}{2}$$

*D*_V_ includes both the crack width and the elastic deformation of the concrete matrix under load. Rastiello et al. [35] found that the two parts of concrete after cracking under a splitting tensile loading can still be considered as elastic bodies. Therefore, the *D*_COD_ is calculated using Equation (2).

(2) $$D_{\mathrm{COD}}=D_{V}-\Delta_{d,e}$$

Δ_d,e_ represents the elastic deformation of the concrete in the radial direction, which can be calculated based on the load–*D*_V_ curve. Equation (2) establishes the relationship between the *D*_V_ and the *D*_COD_ of the concrete specimen.

Akhavan et al. [8] found that, in order to accurately measure the crack permeability, it is necessary to obtain the effective crack width (*b*) [26]. First, the crack area on the specimen surface is required. However, as the crack on the specimen surface was covered with the water vessels, rubber rings and waterproof tape during the permeability test, it is difficult to capture the crack patterns on the specimen surface. Nonetheless, the *D*_V_ of the concrete specimen under splitting tensile load could be precisely measured. Therefore, the effective crack area (*A^eff^*) was indirectly determined by the *D*_COD_ during the permeability test, based on the statistical relationship between the *D*_V_ and *D*_COD_.

**Figure 4:**
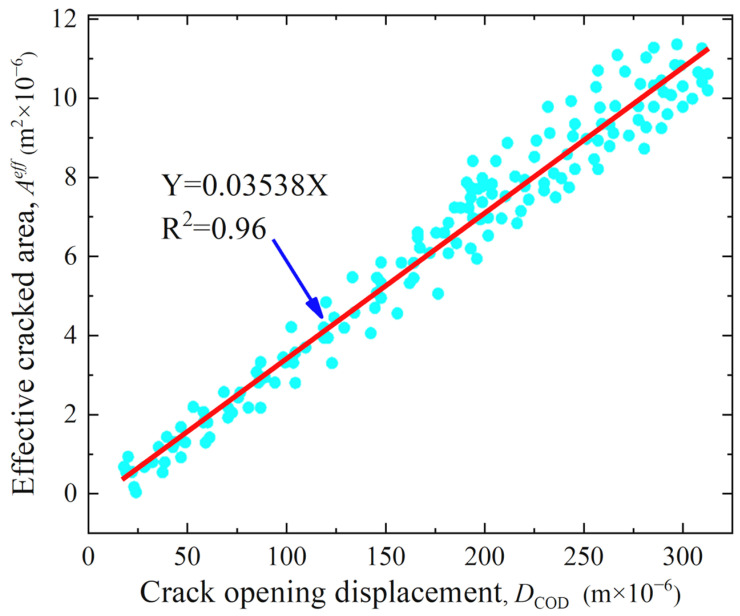
Statistical relationship between *D*_COD_ and *A^eff^* of representative concrete specimen.

Figure 4 shows the data of *D*_COD_ and corresponding to *A^eff^* of a representative concrete specimen and demonstrates the statistical relationship between the *D*_COD_ and *A^eff^*. From Figure 4, the *D*_COD_ and *A^eff^* have a good linear relationship, which can be expressed by Equation (3).

(3) Y=c⋅X

where parameter *c* is the fitting parameter of the statistical relationship between the *D*_COD_ and *A^eff^*.

With the increasing of the *D*_COD_ of the specimen under loading, the *A^eff^* on the surface of the concrete specimen also increases linearly. Using the relationship between the *D*_COD_ and *A^eff^* of the concrete specimen, the *A^eff^* on the concrete surface covered with water vessels in the permeability test can be indirectly evaluated by the *D*_COD_. The fitting parameter *c* and the correlation coefficient *R*^2^ of the relationship between the *D*_COD_ and *A^eff^* for each group of specimens are listed in Table 3. The freeze–thaw cycles of the specimens were 0, 50, 100 and 150, respectively. From Table 3, the fitting parameter *c* of the statistical relationship between the *D*_COD_ and *A^eff^* of different specimens ranges between 0.02773 and 0.0564, with a correlation coefficient of *R*^2^ ≥ 0.91. This implies that the *A^eff^* can be determined from the *D*_COD_ using the statistical relationship between the *D*_COD_ and *A^eff^*.

### 2.4. Permeability Test

**Figure 5:**
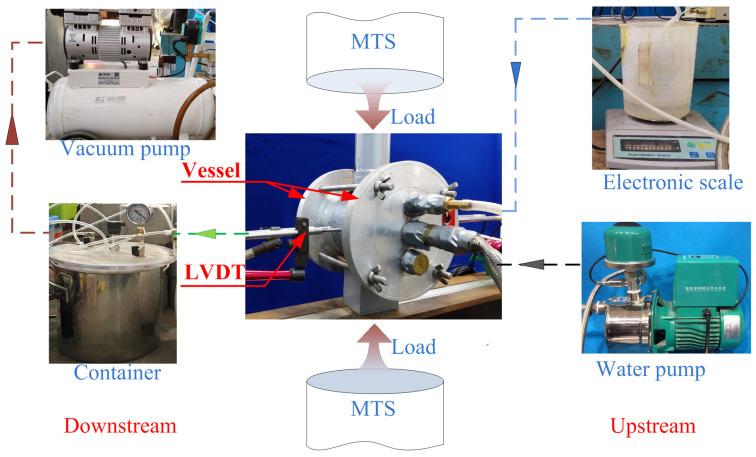
Schematic view of permeability setup.

As shown in Figure 5, the device includes a vacuum pump, a water pump, an electronic scale, a container and two water vessels. The principle of the device is to fill the water vessel in the upstream with water using the water pump, and to decrease the air pressure in the downstream using the vacuum pump; a pressure difference of Δ*p* = 90 kPa from the upstream to the downstream of the permeability setup was created. An electronic scale with an accuracy of 0.0001 g was situated at the upstream of the permeability setup, and a beaker with water was placed on the electronic scale. The pressure difference caused the water from the beaker to flow through the concrete specimen and finally into the container. When the test started, the decrease in mass on the electronic scale was the mass of water that flowed through the specimen. This enabled a real-time measurement of the mass and evaluation of the permeability of the concrete under loading.

During the permeability test, when cracks appear in the concrete specimens, water permeates through both the concrete cracks and the concrete matrix [36]. Consequently, the water flow rate (*Q*) included the water flow through the concrete crack (*Q*_c_) and through the concrete matrix (*Q*_m_). Seong et al. [37] found that the splitting tensile load does not affect the *Q*_m_ of the concrete. Therefore, the *Q*_m_ can be obtained from the permeability test without a splitting tensile load at the beginning of the test. The *Q*_c_ was calculated by Equation (4).

(4) $$Q_{c}=Q-Q_{m}$$

**Figure 6:**
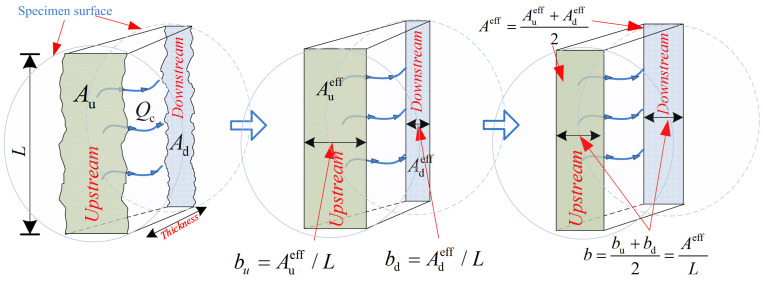
Effective crack width of concrete, where *A*_u/d_ is the crack area on both concrete sides, m^2^; *A*_u/d_^eff^ is the effective crack area on the concrete surface, m^2^; and *b*_u/d_ is the effective crack width on both concrete sides, m.

During permeability tests, the hydraulic pressure gradient was kept below 1.8 MPa/m to ensure laminar water flow into the concrete specimens. Darcy’s law could be used to evaluate the crack permeability [35], represented by Equation (5).

(5) $$\kappa_{c}=\frac{Q_{c}\mu }{A^{eff}}{\left(\frac{\Delta p}{\Delta x}\right)}^{-1}=\frac{Q_{c}\mu }{b\cdot L}{\left(\frac{\Delta p}{\Delta x}\right)}^{-1}$$

where *κ_c_* represents the crack permeability, m^2^; Δ*p*/Δ*x* specifies the hydraulic pressure gradient, Pa/m; *ρ* is the density of water at room temperature, kg/m^3^; *A^eff^* is the crack effective area, m^2^; *μ* is the dynamic viscosity of water, 0.001 Pa·s; *L* represents the crack length oriented perpendicular to the water flow direction; and *b* is the effective crack width, m, as shown in Figure 6.

### 2.5. Collection of Crack Surface Topography

**Figure 7:**
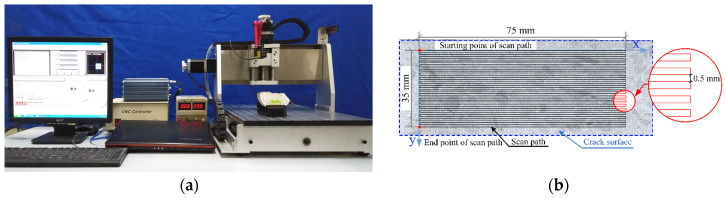
(**a**) Scanning of crack surface of specimen, (**b**) scanning path of crack surface.

Following the permeability test, the specimen was bisected along the crack. The fibers on the crack surface of the specimen were cut, and a laser scanner was used to perform morphological scanning and information collection of the crack surface. The scanning area was 75 mm × 35 mm on the crack surface, as shown in Figure 7. The crack surface roughness *R*_n_ of the concrete specimen was calculated by Equation (6).

(6) $$R_{n}=\frac{S_{t}}{S_{o}}$$

where *S*_o_ is the projected area of the scanning region, 75 mm × 35 mm; *S*_t_ is the crack surface area of the scanning region, mm^2^.

## 3. Results and Discussion

### 3.1. Crack Permeability of Concrete

**Figure 8:**
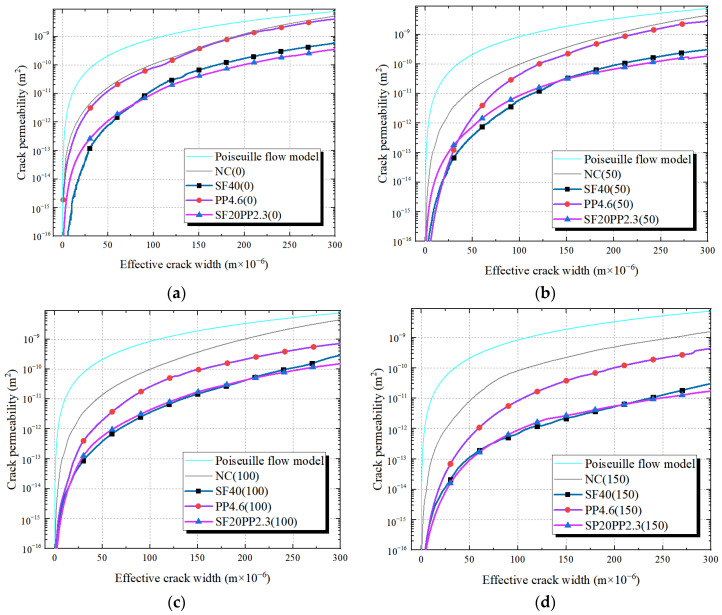
Relationship of the effective crack width and the crack permeability of SF20PP2.3, SF40 and PP4.6 exposed to various freeze–thaw cycles: (**a**) 0 freeze–thaw cycle; (**b**) 50 freeze–thaw cycles; (**c**) 100 freeze–thaw cycles; (**d**) 150 freeze–thaw cycles.

**Figure 9:**
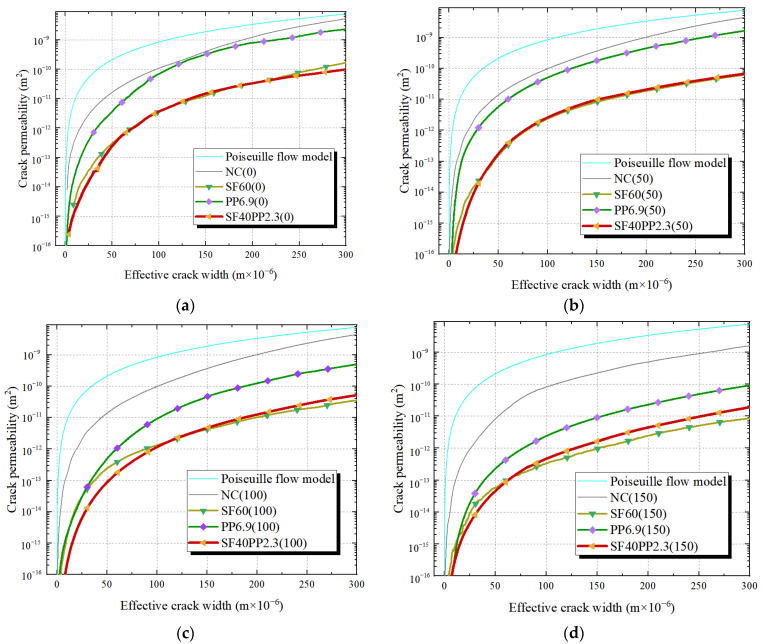
Relationship of the effective crack width and the crack permeability of SF40PP2.3, SF60 and PP6.9 exposed to various freeze–thaw cycles: (**a**) 0 freeze–thaw cycle; (**b**) 50 freeze–thaw cycles; (**c**) 100 freeze–thaw cycles; (**d**) 150 freeze–thaw cycles.

Figure 8 and Figure 9 present the effective crack width–crack permeability curves of the HFRC specimens with a fiber volume content of 0.5 vol.% and 0.75 vol.%, respectively. The mono FRC specimens with the same fiber volume content and the Poiseuille flow model serve as references. As the effective crack width values are 100 μm and 200 μm for the specimens with a fiber volume content of 0.5 vol.% and 0.75 vol.%, respectively, the crack permeability values *κ_c_*_-100_ and *κ_c_*_-200_ are shown in Table 4 and Table 5.

From Figure 8 and Table 4:

Compared to specimens with a fiber volume content of 0.5 vol% (SF20PP2.3, SF40 and PP4.6), it can be observed that when the freeze–thaw cycles range from 0 to 150, the SF20PP2.3 and SF40 specimens exhibit similar crack permeability–effective crack width curves and a lower crack permeability than that of PP4.6.

When the freeze–thaw cycles are the same, SF20PP2.3 exhibits a higher crack permeability than that of SF40 for an effective crack width less than 50 μm. However, as the crack of the concrete widens, the increased rate of crack permeability of SF20PP2.3 is less than that of SF40. For example, when the freeze–thaw cycles are 50, as the effective crack width increases from 100 μm to 200 μm, the crack permeability values of SF40 and SF20PP2.3 increase by 13.5 and 6.7 times, respectively. Hence, the crack permeability value of SF40 is equal to that of SF20PP2.3 for certain effective crack widths. As shown in Figure 8, when the freeze–thaw cycles are 0, 50, 100 and 150, the same crack permeability values for SF20PP2.3 and SF40 are found at an effective crack width of 76 μm, 148 μm, 186 μm and 212 μm, respectively. It is evident that, as the freeze–thaw cycles increase, the effective crack width of equal crack permeability values of SF20PP2.3 and SF40 increases. Moreover, as the crack continues to expand, the crack permeability of SF20PP2.3 remains lower than that of SF40.

From the analysis above, it is evident that, when the effective crack width is small, the crack permeability of SF20PP2.3 is higher than that of SF40. As the crack width increases, the crack permeability of SF20PP2.3 gradually becomes lower than that of SF40 and shows the superior impermeability performance.

From Figure 9 and Table 5:

Compared to specimens with a fiber volume fraction of 0.75 vol% (SF40PP2.3, SF60 and PP6.9), it is seen that, when freeze–thaw cycles range from 0 to 150, SF40PP2.3 and SF60 show similar crack permeability–effective crack width curves and a lower crack permeability than that of PP6.9. When the effective crack width is small, the crack permeability of SF40PP2.3 is less than that of SF60. As the effective crack width widens, the increase rate of crack permeability of SF40PP2.3 exceeds that of SF60. Consequently, the crack permeability of SF60 becomes equal to that of SF40PP2.3 for certain effective crack widths. For the specimens without freeze–thaw damage, the permeability value of SF40PP2.3 is found to be lower than that of SF60. When the freeze–thaw cycles reach 50, 100 and 150, the same crack permeability values for SF40PP2.3 and SF60 are observed at an effective crack width of 36 μm, 131 μm and 62 μm, respectively. As the effective crack width continues to expand, the permeability of SF40PP2.3 becomes greater than that of SF60. This indicates that SF40PP2.3 with hybrid fibers exhibits a higher crack impermeability than that of mono FRC, when the concrete structure works in an environment without freeze–thaw cycles. For SF40PP2.3 and SF60 with freeze–thaw damage, with the increasing of the effective crack width, the advantage of hybrid fibers in enhancing concrete crack impermeability gradually diminishes and disappears, and SF60 shows better crack impermeability than SF40PP2.3.

### 3.2. Permeability Performance Evaluation of FRC

When the crack surfaces are parallel and absolutely smooth, the permeability can be estimated by the Poiseuille flow model (*κ_PFM_*), as shown in Equation (7).

(7) $$\kappa_{PFM}=\frac{{b_{p}}^{2}}{12}$$

where, *b_p_* is the distance between parallel plates of the Poiseuille flow model. 

However, researchers have found that cracks in cementitious materials are rough and tortuous, which does not align with the conditions of the Poiseuille flow model [8,26,35]. In order to assess the crack permeability of concrete using the Poiseuille flow model, some researchers [8,38] have introduced a modified factor *ξ* to quantify the influence of crack surfaces on the crack permeability of cracked concrete. The modified factor *ξ* is calculated by Equations (8) and (9):

(8) $$\kappa_{c}=\xi \frac{{b_{p}}^{2}}{12}$$

(9) $$\xi =\frac{\kappa_{c}}{\kappa_{PFM}}$$

**Figure 10:**
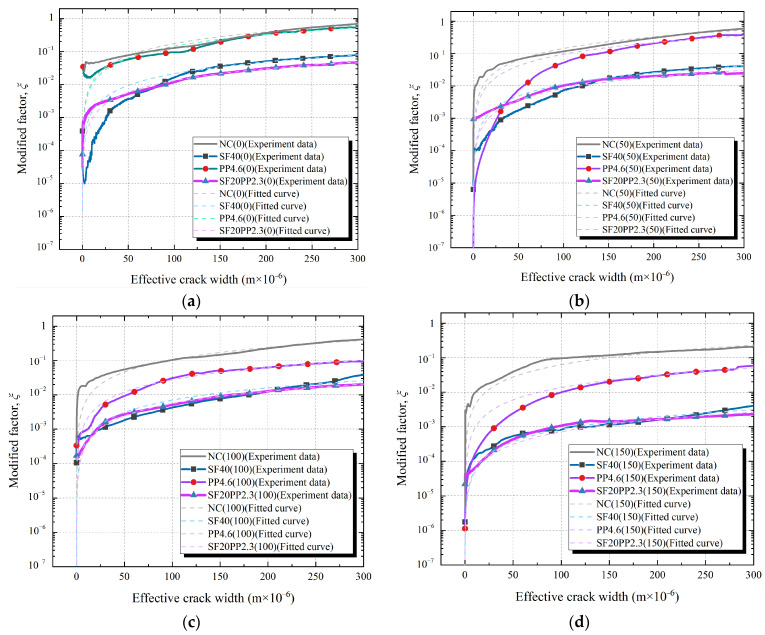
Relationship of the effective crack width and the modified factor *ξ* of SF20PP2.3, SF40 and PP4.6 exposed to freeze–thaw cycles: (**a**) 0 freeze–thaw cycle; (**b**) 50 freeze–thaw cycles; (**c**) 100 freeze–thaw cycles; (**d**) 150 freeze–thaw cycles.

**Figure 11:**
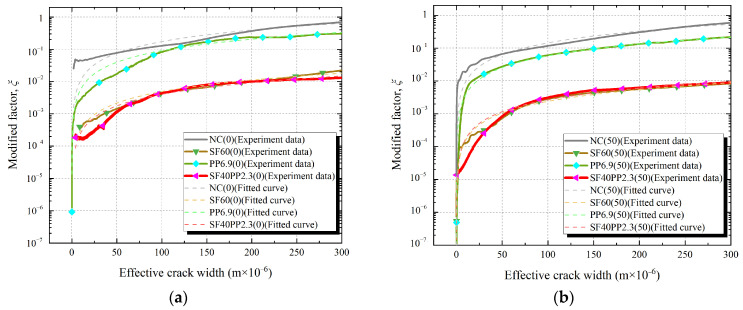

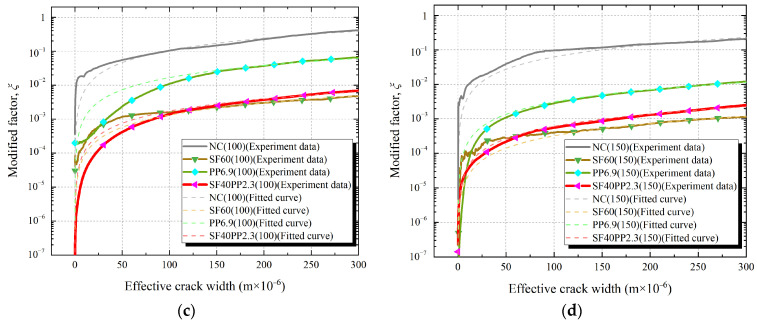
Relationship of the effective crack width and the modified factor *ξ* of SF40PP2.3, SF60 and PP6.9 exposed to freeze–thaw cycles: (**a**) 0 freeze–thaw cycle; (**b**) 50 freeze–thaw cycles; (**c**) 100 freeze–thaw cycles; (**d**) 150 freeze–thaw cycles.

Figure 10 and Figure 11 show the relationships of the modified factor *ξ* and the effective crack width of specimens with a fiber volume content of 0.5 vol.% and 0.75 vol.%, respectively.

From Figure 10 and Figure 11, the following can be seen:

(1) When the effective crack width of the specimens is ≤25 μm, the modified factor *ξ* exhibits noticeable fluctuations with the increase in the effective crack width. This phenomenon gradually disappears as the effective crack width increases. This can be attributed to the small crack width and incomplete separation of the crack surface (see Table 2) at the initial stage of crack formation. As the splitting tensile load increases and the crack widens, the aggregates on the crack surface may interlock. This causes the phenomenon of the “widening-closing-rewidening” of the local crack width and results in significant fluctuations in the effective crack width-modified factor *ξ* curves. A similar phenomenon has been confirmed in the literature [35]. As the crack width of the specimens continues to increase, the crack surfaces fully separate, and the fluctuations in the effective crack width-modified factor *ξ* curves gradually diminish.
(2) With the increasing of the effective crack width of the specimens, the crack permeability of each specimen is closer to the *κ*_PFM_. This is because the crack permeability of concrete is primarily determined by two factors: crack width and crack surface roughness. According to the Poiseuille flow model, permeability is directly proportional to the square of the distance between the parallel surfaces. This suggests that when the effective crack width of concrete increases, a significant increase in crack permeability may occur. For the concrete crack, the rough crack surface leads to significant reductions in crack permeability [26]. However, when the crack surfaces are fully separated, the roughness of the crack surface remains almost constant. There is no significant change in the effect of nearly constant surface roughness on crack permeability. Therefore, the crack permeability of concrete becomes high, and closer to the *κ*_PFM_, with the increasing of the effective crack width. Akhavan et al. [8] and Rastiello et al. [35] have shown similar results in studying the permeability of cracked concrete.
(3) For HFRC specimens, the modified factor *ξ* of crack permeability is observed to gradually increase with the expansion of the concrete cracks. When the fiber volume content and freeze–thaw cycles of the specimens are the same, the *ξ*-*ω* curves of the HFRC specimens are found to be closer to those of the SFRC specimens. This is similar to the trend observed in their crack permeability–effective crack width curves.

In previous studies [8,38], the modified factor *ξ* was often regarded as a constant to estimate the permeability of cracked concrete. However, the modified factor *ξ* is observed to gradually increase with crack propagation. Therefore, it can only serve as an evaluation parameter of crack permeability performance with a specific crack width and cannot characterize the overall trend of crack permeability performance with crack propagation. In order to accurately estimate the effect of hybrid fibers on concrete crack permeability, this study incorporated the prediction model of the Poiseuille flow model and derived the permeability parameter *α* to evaluate crack permeability development trends in cracked concrete, as shown in Equations (10) and (11).

(10) $$\xi =\xi (\omega )=\alpha \omega^{\beta }$$

(11) $$\kappa_{c}=\xi \cdot \kappa_{p}=\xi (\omega )\cdot \kappa_{p}=\alpha \omega^{\beta }\cdot \kappa_{p}=\frac{\alpha \omega^{\beta +2}}{12}$$

where *α* is the permeability parameter and *β* is the constant factor of the fitted equation.

When the effective crack width and *β* are constant, the permeability parameter *α* exhibits a direct linear correlation with the crack permeability. Additionally, the permeability parameter *α* can characterize the development trend of crack permeability within a certain range of crack widths. Therefore, compared to the modified factor *ξ*, the permeability parameter *α* is suitable to assess the effect of hybrid fibers on the crack permeability of cracked concrete.

**Figure 12:**
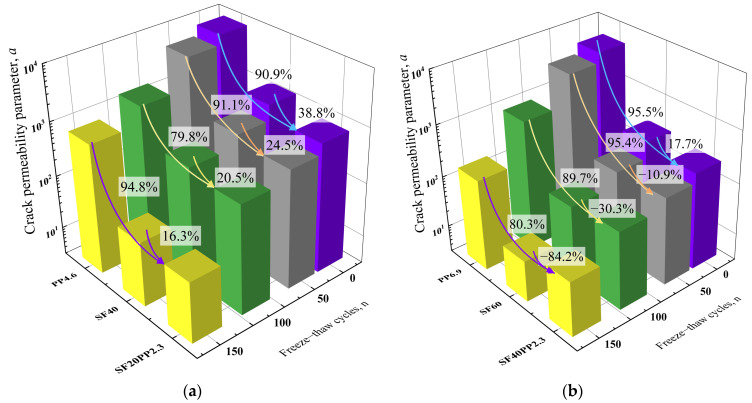
Bar charts of permeability parameter *α*: (**a**) specimen with a fiber volume content of 0.5 vol.%; (**b**) specimen with a fiber volume content of 0.75 vol.%.

To estimate the hybrid fibers’ influence on the evolution trend of crack permeability, a linear fit between the modified factor *ξ* and the effective crack width of the concrete specimens is performed by Equation (11). The value of parameter *β* is 1.17 [35]. The fitted curve of each specimen is shown in Figure 10 and Figure 11. The permeability parameter *α* values are obtained from the linear fit and are presented in Table 6. The bar chart of the permeability parameter *α* of each specimen group is shown in Figure 12.

From Table 6 and Figure 12a, compared to PP4.6, the permeability parameter *α* of SF20PP2.3 subjected to 0, 50, 100 and 150 freeze–thaw cycles decreases by 90.9%, 91.1%, 79.8% and 94.8%, respectively. In contrast, compared with SF40, the permeability parameter *α* of SF20PP2.3 subjected to 0, 50, 100 and 150 freeze–thaw cycles decreases by 38.8%, 24.5%, 20.5% and 16.3%, respectively. Therefore, based on an analysis using permeability parameter *α*, SF20PP2.3 with hybrid fibers demonstrates a significant advantage in improving crack impermeability.

From Table 6 and Figure 12b, compared to PP6.9 exposed to 0, 50, 100 and 150 freeze–thaw cycles, the permeability parameter *α* of SF40PP2.3 decreases by 95.5%, 95.4%, 89.7% and 80.3%, respectively. Compared to SF60 without freeze–thaw damage, the permeability parameter *α* of SF40PP2.3 decreases by 17.7%, and compared to SF60 exposed to 50, 100 and 150 freeze–thaw cycles, the permeability parameter *α* of SF40PP2.3 increases by 10.9%, 30.3% and 84.2%, respectively. An analysis based on permeability parameter *α* indicates that SF40PP2.3 without freeze–thaw damage exhibits a higher crack impermeability than mono FRC with the same fiber volume content. However, as the freeze–thaw cycles increase, the permeability parameter *α* of SF40PP2.3 is lower than that of PP6.9 but higher than that of SF60. Therefore, the crack impermeability of SF40PP2.3 lies between that of the PP6.9 and SF60, subjected to the same freeze–thaw cycles.

### 3.3. Morphological Analysis of Crack Surface

**Figure 13:**
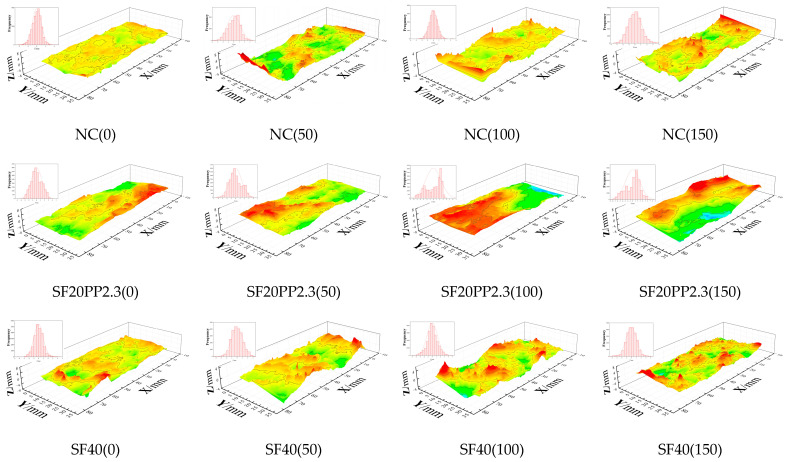

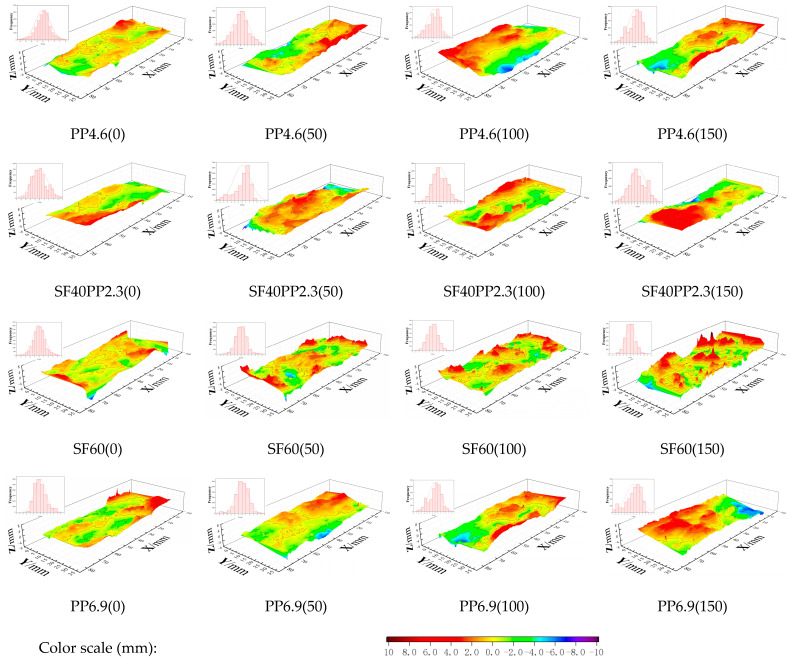
Reconstruction images of crack surface.

Crack surfaces of specimens from each group are scanned for morphological analysis using the laser-scanning setup. Crack surface morphology data for each specimen are processed with Origin software to reconstruct 3D graphics of the crack surface. A consistent color scale is used for the 3D reconstructions of all specimen groups. The 3D reconstruction images of the crack surfaces of HFRC are shown in Figure 13, and the mono FRC specimens with the same fiber volume content and NC specimens are the reference. The crack surface roughness (*R*_n_) of each group is listed in Table 7.

From Figure 13 and Table 7, the following can be seen:

(1) When the specimens are exposed to the same freeze–thaw cycles, the crack surface of FRC is rougher than that of NC.
(2) The crack surface *R*_n_ values of all groups increase with the increment in freeze–thaw cycles.
(3) When the addition of fiber volume content is 0.5 vol.%, the crack surface *R*_n_ values of SF20PP2.3 consistently exceed those of SF40 and PP4.6 exposed to 0, 50, 100 and 150 freeze–thaw cycles.
(4) When the fiber volume content of the specimens is 0.75 vol.%, the crack surface *R*_n_ value of SF40PP2.3 without freeze–thaw damage is consistently higher than those of SF60 and PP6.9. However, with the increment in freeze–thaw cycles, the crack surface *R*_n_ value of SF40PP2.3 becomes higher than that of SF60 but lower than that of PP6.9.

**Figure 14:**
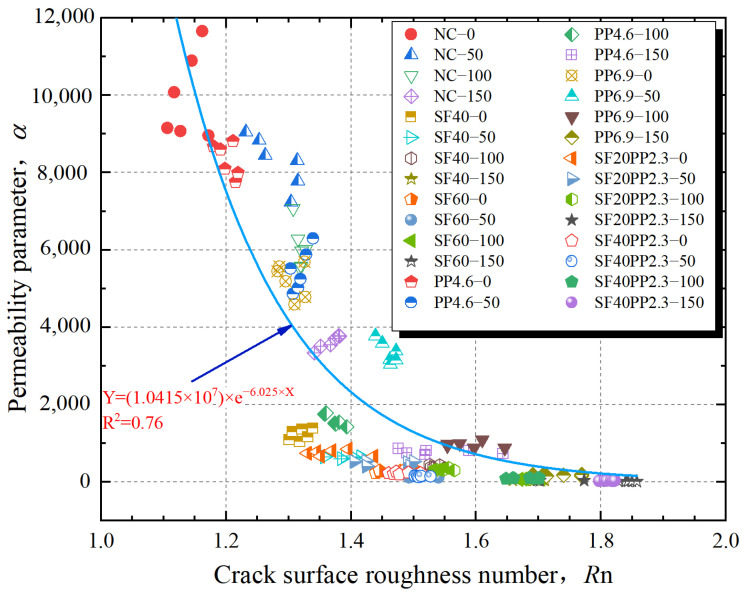
Relationship between crack surface *R*_n_ and permeability parameter *α* of cracked concrete.

From the discussion above, it is concluded that an increase in crack surface roughness correlates with a gradual decrease in crack permeability. A similar phenomenon has been observed in previous studies [3,39]. To estimate the relationship between crack permeability and the crack surface morphology, the relationship between the crack surface *R*_n_ and permeability parameter *α* of different specimens is illustrated in Figure 14.

From Figure 14, it is apparent that an exponential functional relationship exists between the permeability parameter *α* and the crack surface *R*_n_, which can be expressed by Equation (12). The fitted parameters are presented in Table 8.

(12) $$Y=\gamma \cdot e^{\left(\tau \cdot X\right)}$$

where *γ* and *τ* are the parameters obtained through fitting experimental data.

The correlation coefficient *R*^2^ of the *α*–*R*_n_ curve is 0.76; a similar phenomenon was confirmed in the literature [26]. This indicates a significant correlation between the crack permeability and the roughness of the crack surface. Specifically, the rougher the surface of the crack, the lower its crack permeability is. Therefore, the crack impermeability of FRC can be characterized by its crack surface roughness. This also demonstrates that HFRC specimens can effectively increase the crack surface roughness and enhance the crack impermeability of concrete.

### 3.4. Analysis of Positive Synergistic Effect of Hybrid Fibers on Crack Impermeability

**Figure 15:**
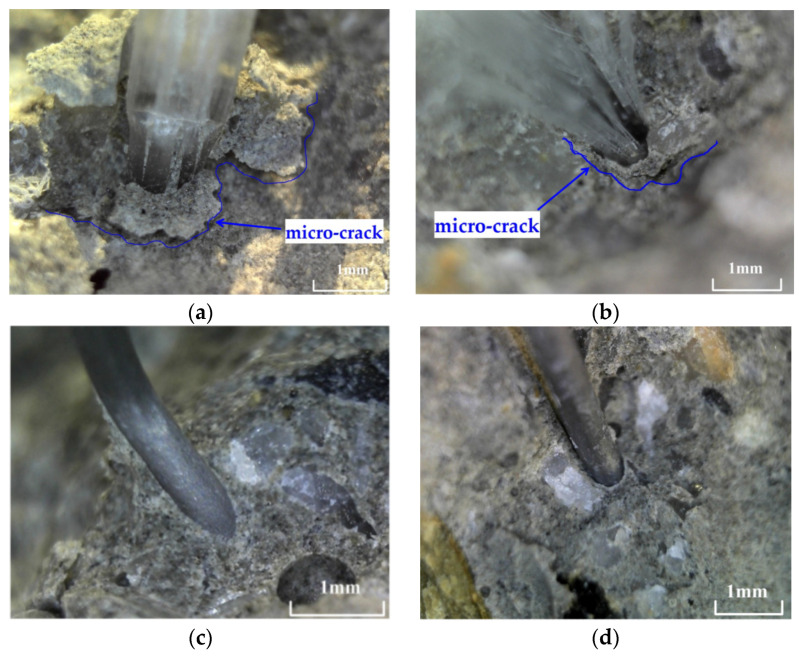
Interface zone between fiber and concrete matrix: (**a**,**b**) polypropylene fiber and concrete matrix; (**c**,**d**) steel fiber and concrete matrix.

To investigate the effects of polypropylene fibers and steel fibers on crack formation in concrete reinforced with both types of fibers, the main crack in each group of specimens is propagated. Figure 15a,b illustrate the interface zone between polypropylene fibers and the concrete matrix on the crack surface. The blue line shows the micro-crack between the polypropylene fiber and the concrete matrix. Figure 15c,d illustrate the interface zone between the steel fibers and the concrete matrix on the crack surface.

From Figure 15, a micro-crack can be observed at the interface zone between the polypropylene fiber and the concrete matrix. However, the interface zone between steel fiber and concrete matrix is sound. This implies that the anchorage of the steel fibers with the concrete matrix is higher than that of the polypropylene fibers. De Alencar Monteiro et al. [40] and Biao et al. [41] have shown similar results in their studies on the mechanical behavior of SFRC and PFRC. The interface zone between the polypropylene fiber and concrete matrix is the weak area of concrete.

**Figure 16:**
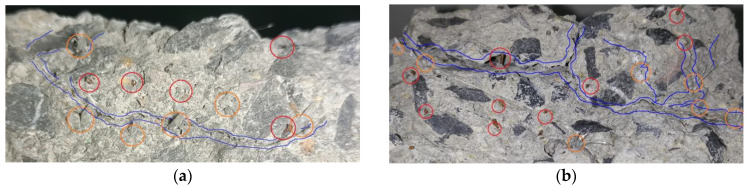

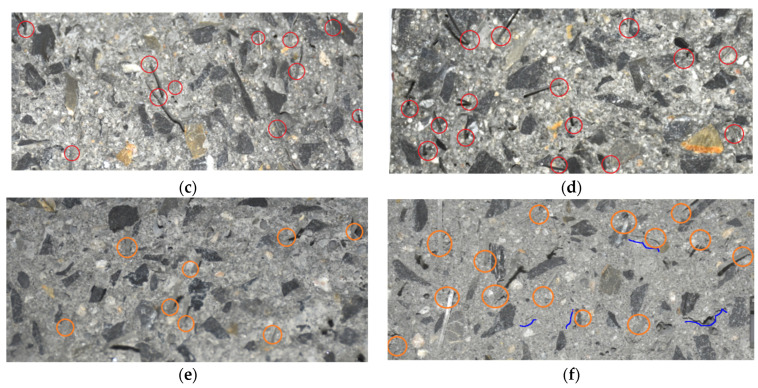
Crack surface topographies of (**a**) SF20PP2.3, (**b**) SF40PP2.3, (**c**) SF40, (**d**) SF60, (**e**) PP4.6 and (**f**) PP6.9.

Figure 16 shows the crack surface topographies of HFRC, SFRC and PFRC specimens, respectively. The blue lines represent micro-cracks on the crack surface, the red circles represent steel fibers, and the yellow circles represent polypropylene fibers.

From Figure 16, it is evident that HFRC (SF20PP2.3 and SF40PP2.3) specimens are more prone to micro-crack formation than SFRC (SF40 and SF60) and PFRC (PP4.6 and PP6.9) specimens. Moreover, at the locations of micro-cracks, polypropylene fibers can be observed to be distributed along the direction of micro-crack propagation (blue lines), while many steel fibers are embedded in the concrete matrix and bridge the micro-cracks. In contrast, the main crack surfaces of mono FRC specimens show no micro-cracks. This phenomenon may be attributed to the high elastic modulus and hooked ends of steel fibers, which provide a high anchorage between steel fibers and the concrete matrix. In comparison, polypropylene fibers, with a low elastic modulus and straight ends, show weak anchorage between the polypropylene fibers and concrete matrix. The polypropylene fibers represent the weak area of concrete in the concrete matrix. As the concrete is loaded, the interface zone between the polypropylene fibers and concrete matrix is more prone to form micro-cracks than the concrete matrix.

For HFRC specimens with polypropylene fibers and steel fibers, the steel fibers bear tensile stress on both crack faces and effectively transmit the stress into the concrete matrix, while polypropylene fibers induce the formation of micro-cracks. The synergistic action of the two types of fibers promotes the formation of micro-cracks in the concrete matrix and leads to the propagation of micro-cracks into macro-cracks in the concrete. Therefore, micro-cracks increase the total surface area of concrete cracks. A large crack surface area effectively increases the actual path length for water flow through the concrete specimens. This results in the increased head loss and improved crack impermeability of the cracked concrete. Moreover, compared to mono FRC (SFRC and PFRC), HFRC is more prone to both micro-cracks and macro-cracks. This is one of the key factors of the positive synergistic effect on the crack impermeability of cracked concrete.

## 4. Conclusions

This study investigated hybrid fiber’s influence on the crack permeability of cracked concrete exposed to freeze–thaw cycles. The experimental and analytical results led to the following conclusions:

(1) The modified factor *ξ* of crack permeability gradually increases and is closer to 1 with the expansion of the concrete crack. When the effective crack width of the specimens is less than 25 μm, the two sides of the crack surface are not completely separated and the “widening-closing-widening” of the local crack width significantly reduces the crack permeability. When the effective crack width of the specimens is beyond 25 μm, the crack surfaces are completely separated and the roughness of the crack surface remains almost unchanged. The effect of crack surface roughness on crack permeability gradually decreases, and the crack permeability of each specimen is closer to the *κ*_PFM_.
(2) Compared with the modified factor *ξ* of crack permeability, the permeability parameter *α* can effectively evaluate and quantify the development trend of crack permeability within a certain range of crack widths and be used as a quantitative parameter for the durability design of concrete materials.
(3) For specimens with a fiber volume content of 0.5 vol.%, compared to PP4.6 and SF40, the permeability parameter α of SF20PP2.3, subjected to the same freeze–thaw cycles, decreases by 16.3–94.8%. SF20PP2.3 demonstrates a positive synergistic effect on the crack impermeability of cracked concrete. For specimens with a fiber volume content of 0.75 vol.%, compared to PP6.9 and SF60, the permeability parameter α of SF20PP2.3 without freeze–thaw damage decreases by 17.7–95.5%, and the crack impermeability of SF40PP2.3 subjected to the same freeze–thaw cycles lies between that of PP6.9 and SF60.
(4) The roughness of the crack surface and the crack permeability is highly correlated and follows an exponential curve (Y = 1.0415 × 10^7^·e^−6.025·X^, Y is the crack permeability parameter *α*, X is the crack surface *R*_n_) in concrete. This indicates that hybrid fibers enhance crack impermeability by increasing the crack surface roughness. Based on this relationship, the permeability of cracked concrete can be quickly evaluated by crack surface roughness in real engineering applications.
(5) The combination of steel fibers and polypropylene fibers effectively promotes the formation of micro-cracks in the concrete matrix. This increases the actual path length of water flow through concrete specimens and enhances the hydraulic head loss. This is a key reason for the evident positive synergistic effect on the crack impermeability of cracked concrete.

## Figures and Tables

**Table 1:** Fiber content of specimens.

Mixture ID	Macro Fiber Volume Content	Steel Fiber	Polypropylene Fiber
NC	0 vol.%	0 kg/m^3^ (0 vol.%)	0 kg/m^3^ (0 vol.%)
SF20PP2.3	0.50 vol.%	20 kg/m^3^ (0.25 vol.%)	2.3 kg/m^3^ (0.25 vol.%)
SF40		40 kg/m^3^ (0.5 vol.%)	0 kg/m^3^ (0 vol.%)
PP4.6		0 kg/m^3^ (0 vol.%)	4.6 kg/m^3^ (0.5 vol.%)
SF40PP2.3	0.75 vol.%	40 kg/m^3^ (0.5 vol.%)	2.3 kg/m^3^ (0.25 vol.%)
SF60		60 kg/m^3^ (0.75 vol.%)	0 kg/m^3^ (0 vol.%)
PP6.9		0 kg/m^3^ (0 vol.%)	6.9 kg/m^3^ (0.75 vol.%)

**Table 2:** Crack evolution process.

*D*_COD_	Morphology of Crack on the Specimen Surface
10 μm	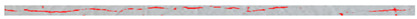
50 μm	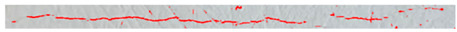
100 μm	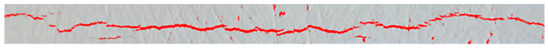
200 μm	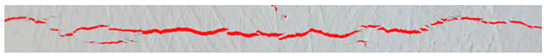
300 μm	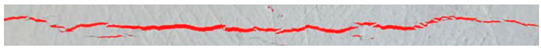

**Table 3:** Fitting parameter *c* between *D*_COD_ and *A^eff^* of different specimens.

Freeze–Thaw Cycles	Fitting Parameter *c*						
	NC	SF20PP2.3	SF40	PP4.6	SF40PP2.3	SF60	PP6.9
0	0.02906	0.0369	0.03538	0.0441	0.0348	0.04039	0.0424
	(*R*^2^ = 0.99)	(*R*^2^ = 0.91)	(*R*^2^ = 0.96)	(*R*^2^ = 0.95)	(*R*^2^ = 0.98)	(*R*^2^ = 0.98)	(*R*^2^ = 0.97)
50	0.02773	0.0471	0.05042	0.0492	0.0564	0.03383	0.0475
	(*R*^2^ = 0.97)	(*R*^2^ = 0.99)	(*R*^2^ = 0.99)	(*R*^2^ = 0.97)	(*R*^2^ = 0.96)	(*R*^2^ = 0.97)	*R*^2^ = 0.96)
100	0.04719	0.0512	0.03343	0.0358	0.0429	0.05608	0.0511
	(*R*^2^ = 0.95)	(*R*^2^ = 0.98)	(*R*^2^ = 0.94)	(*R*^2^ = 0.97)	(*R*^2^ = 0.99)	(*R*^2^ = 0.99)	(*R*^2^ = 0.99)
150	0.03472	0.0461	0.04161	0.0377	0.0476	0.04079	0.0294
	(*R*^2^ = 0.96)	(*R*^2^ = 0.97)	(*R*^2^ = 0.98)	(*R*^2^ = 0.96)	(*R*^2^ = 0.99)	(*R*^2^ = 0.98)	(*R*^2^ = 0.99)

**Table 4:** Comparison of crack permeability of SF20PP2.3, SF40 and PP4.6 exposed to various freeze–thaw cycles.

Freeze–Thaw Cycles	*κ_c_*_-100_ (m^2^)			*κ_c_*_-200_ (m^2^)		
	SF20PP2.3	SF40	PP4.6	SF20PP2.3	SF40	PP4.6
0	9.97 × 10^−12^	1.38 × 10^−11^	7.94 × 10^−11^	1.03 × 10^−10^	1.68 × 10^−10^	1.14 × 10^−9^
50	8.57 × 10^−12^	6.13 × 10^−12^	4.36 × 10^−11^	6.66 × 10^−11^	8.92 × 10^−11^	6.88 × 10^−10^
100	4.31 × 10^−12^	3.55 × 10^−12^	2.56 × 10^−11^	4.25 × 10^−11^	4.16 × 10^−11^	2.10 × 10^−10^
150	8.92 × 10^−13^	6.84 × 10^−13^	8.27 × 10^−12^	5.49 × 10^−12^	5.27 × 10^−12^	1.01 × 10^−10^

**Table 5:** Comparison of crack permeability of SF40PP2.3, SF60 and PP6.9 exposed to various freeze–thaw cycles.

Freeze–Thaw Cycles	*κ_c_*_-100_ (m^2^)			*κ_c_*_-200_ (m^2^)		
	SF40PP2.3	SF60	PP6.9	SF40PP2.3	SF60	PP6.9
0	3.65 × 10^−12^	3.62 × 10^−12^	6.89 × 10^−11^	3.30 × 10^−11^	3.30 × 10^−11^	7.82 × 10^−10^
50	2.60 × 10^−12^	2.36 × 10^−12^	5.04 × 10^−11^	2.07 × 10^−11^	1.86 × 10^−11^	4.44 × 10^−10^
100	1.16 × 10^−12^	1.33 × 10^−12^	9.10 × 10^−12^	1.24 × 10^−11^	1.01 × 10^−11^	1.23 × 10^−10^
150	4.65 × 10^−13^	3.30 × 10^−13^	2.37 × 10^−12^	4.39 × 10^−12^	2.41 × 10^−12^	2.27 × 10^−11^

**Table 6:** Comparison of permeability parameter *α* of different specimens.

Freeze–Thaw Cycles	Permeability Parameter *α*						
	NC	SF20PP2.3	SF40	PP4.6	SF40PP2.3	SF60	PP6.9
0	9971	754	1233	8324	232	282	5213
50	8281	487	645	5473	152	137	3354
100	6078	321	404	1589	99	76	962
150	3608	41	49	784	35	19	178

**Table 7:** Comparison of crack surface *R*_n_ of different specimens.

Freeze–Thaw Cycles	Crack Surface *R*_n_						
	NC	SF20PP2.3	SF40	PP4.6	SF40PP2.3	SF60	PP6.9
0	1.138	1.371	1.319	1.202	1.480	1.466	1.304
50	1.280	1.456	1.395	1.319	1.510	1.511	1.460
100	1.318	1.548	1.529	1.374	1.675	1.680	1.589
150	1.366	1.758	1.692	1.539	1.812	1.838	1.732

**Table 8:** Fitted parameters of an exponential functional relationship.

Curve	*γ*	*τ*	*R* ^2^
*α*–*R*_n_	1.0415 × 10^7^	−6.025	0.76

## Data Availability

Data available on request from the authors. The data that support the findings of this study are available from the corresponding author upon reasonable request.
